# Reconstruction of 3D structures of MET antibodies from electron microscopy 2D class averages

**DOI:** 10.1371/journal.pone.0175758

**Published:** 2017-04-13

**Authors:** Qi Chen, Michal Vieth, David E. Timm, Christine Humblet, Dina Schneidman-Duhovny, Ilan E. Chemmama, Andrej Sali, Wei Zeng, Jirong Lu, Ling Liu

**Affiliations:** 1 Lilly Research Laboratories, Eli Lilly and Company, Indianapolis, Indiana, United States of America; 2 Department of Bioengineering and Therapeutic Sciences, Department of Pharmaceutical Chemistry, and California Institute of Quantitative Biosciences, University of California, San Francisco, San Francisco, California, United States of America; Indian Institute of Science, INDIA

## Abstract

Dynamics of three MET antibody constructs (IgG1, IgG2, and IgG4) and the IgG4-MET antigen complex was investigated by creating their atomic models with an integrative experimental and computational approach. In particular, we used two-dimensional (2D) Electron Microscopy (EM) images, image class averaging, homology modeling, Rapidly exploring Random Tree (RRT) structure sampling, and fitting of models to images, to find the relative orientations of antibody domains that are consistent with the EM images. We revealed that the conformational preferences of the constructs depend on the extent of the hinge flexibility. We also quantified how the MET antigen impacts on the conformational dynamics of IgG4. These observations allow to create testable hypothesis to investigate MET biology. Our protocol may also help describe structural diversity of other antigen systems at approximately 5 Å precision, as quantified by Root-Mean-Square Deviation (RMSD) among good-scoring models.

## Introduction

**Fig 1:**
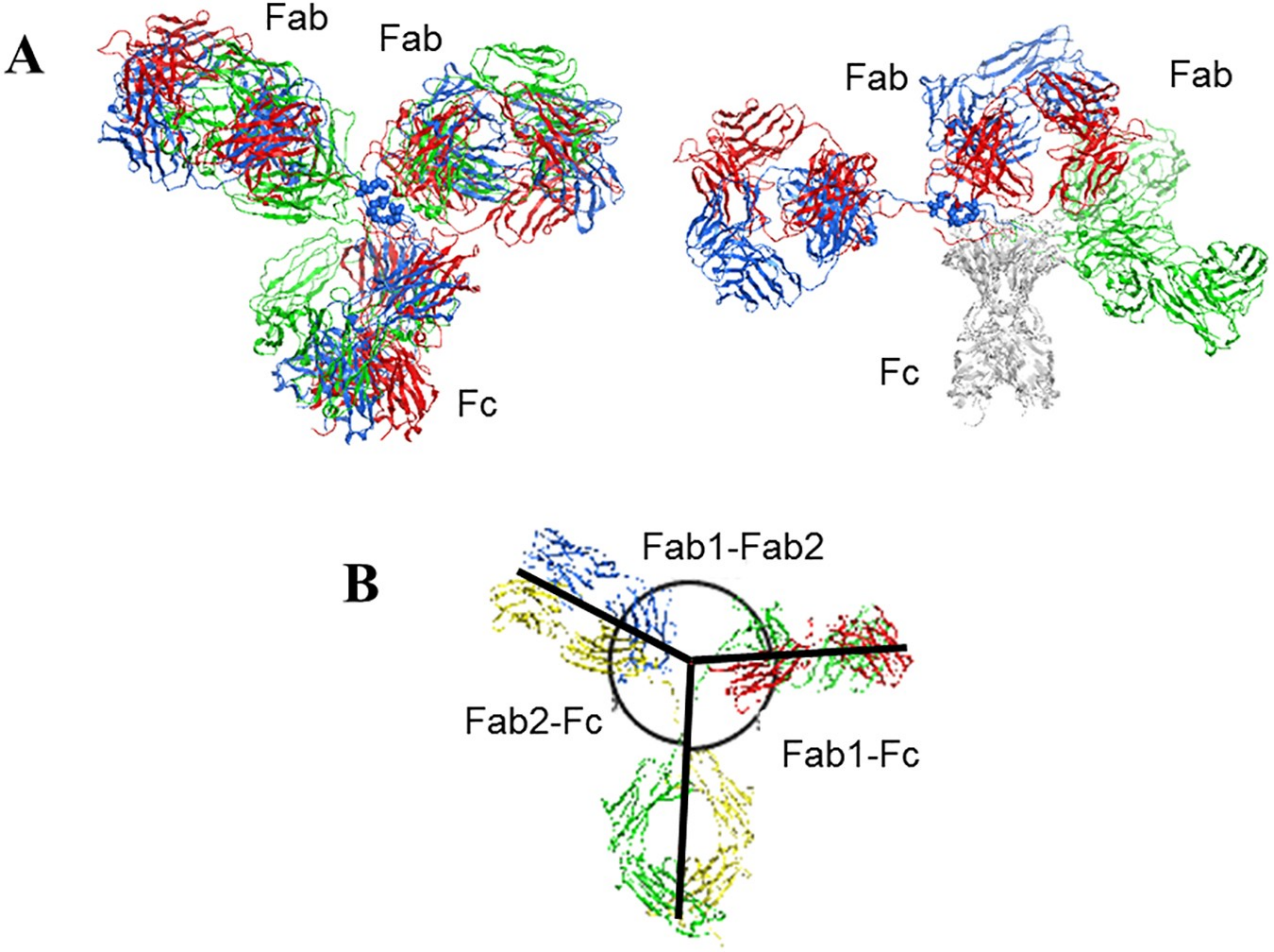
The antibody structure and variability. (A) X-ray crystal structures of three full length antibodies: mouse IgG2 in blue (PDB code 1IGT) [3], human IgG1 in red (1HZH) [4], and mouse IgG1 in green (1IGY) [5], superposed on all aligned C_**α**_ atoms from all three domains (left) for the overall shape comparison or only Fc domains (right) to highlight the differences in Fab domains. The pairwise C_**α**_ RMSD values range from 20 to 34 Å with an average of 27.6 Å. The pairwise C_**α**_ RMSD values of the Fab domains range from 1.1 to 3.9 Å when superposed only on the Fab domain residues, with an average of 2.0 Å. The pairwise C_**α**_ RMSD values of the Fc domains range from 2.2 to 2.4 Å when superposed only on the Fc domain residues. Superposition was done using MOE 2014.09 [6]. (B) An example to show the definition of the domain angles between every two domains measured from 3D structures as described by Zhang et. al. [2]. The lines follow the longest axis of each domain.

Antibodies are among the most specific biomedicines. They are important therapeutic agents, both as biomolecular drugs and as delivery vehicles of drugs in antibody drug conjugates [1]. Antibodies usually contain three domains, i.e., two Fab domains and one Fc domain, connected by two short peptidic hinges (Fig 1). The 3D atomic structure of each full length antibody exists as an ensemble of multiple conformational states [2], although the three domains are almost always arranged into a Y or T-shaped 3D object as shown in their X-ray crystal structures [3–5]. Due to the flexibility of the two hinges, the C_**α**_ RMSD between antibody structures could be higher than 30 Å, despite a similar overall arrangement of the three domains and the structural similarity among the individual Fab and Fc domains (Fig 1). This diverse structural space of antibodies makes the structure determination by X-ray crystallography and the application of structure-based design approaches extremely challenging.

Multiple techniques have been used to study full length antibody structures, including X-ray crystallography that gave the structures of three full length constructs [3–5], 3D Individual Particle Electron Tomography (IPET) [2, 7] and EM imaging [8]. The IPET maps at 10–15 Å resolution combined with molecular dynamics simulations demonstrated a vast structural space represented by 120 diverse structure models [2] available to the mouse IgG1 construct. The model construction in the IPET study used a single starting structure from X-ray crystallography [3], allowing flexibility in the hinge region while keeping the individual domains rigid. The antibody structural space resulting from different arrangements of the rigid domains referred to as the “domain conformations” revealed by the IPET study serves as a starting point for our study.

To model the MET domain conformations, we used the EM2D module [9] of the open source Integrative Modeling Package (IMP) [10, 11] to construct the models of three MET isotypes (IgG1, IgG2, IgG4) from the low resolution (~20 Å, see “Stage 3: Scoring domain conformation” of Materials and Methods section for details) 2D class averages of individual particle EM images. We found that for all examined antibody constructs, every good quality 2D class average could be uniquely represented by a single model of domain conformation selected from a diverse conformational ensemble at model precision of 5 Å RMSD. The variability among the generated models that sufficiently satisfy the experimental 2D class averages is quantified by model precision, defined as the largest RMSD value of a model that still satisfies the 2D class average to the best scoring model for that 2D class average (see “Stage 4: Analysis and Assessment of the Ensemble” in “Materials and Methods” section for details). The determination of domain conformations at 5 Å RMSD precision increases our understanding of antibody structural dynamics. Furthermore, it allows us to relate the biological profile of constructs to the inter-domain interactions, location and orientation of complementarity determining regions (CDRs) as well as the overall shape of the antibodies. The methodology presented here can be applied for future exploration of dynamics of antibodies in general.

Our modeling effort focused on MET antibody constructs. MET, the receptor for hepatocyte growth factor (HGF), has been implicated in driving tumor proliferation and metastasis. Given the critical roles of the MET/HGF pathway in tumor growth and development, various groups developed MET blocking antibodies [12–15]. However, bivalent anti-MET antibodies that inhibit both HGF-dependent and HGF-independent activation were largely unsuccessful as these constructs tended to have agonistic rather than antagonistic activity [13–15]. The first reported construct with no agonistic activity was LY2875358 [12]. LY2875358 is a humanized IgG4 antibody against the MET receptor, currently being evaluated in Phase II clinical trials for non-small cell lung cancer (NSCLC). It has high neutralization and internalization activities, resulting in inhibition of ligand dependent and ligand independent MET pathway inhibition. While LY2875358 does not have agonist activity in the IgG4 format, the IgG1 version of the same MET antibody shows increased agonist activity. This observation suggests that the agonistic activity of the antibody might depend on the IgG isotypes on the top of the differences in the variable regions. Knowledge of the 3D structural differences between different IgG isotypes could help us better understand the above-mentioned functional outcomes.

## Results

### Isotype dependence of agonist activity

**Fig 2:**
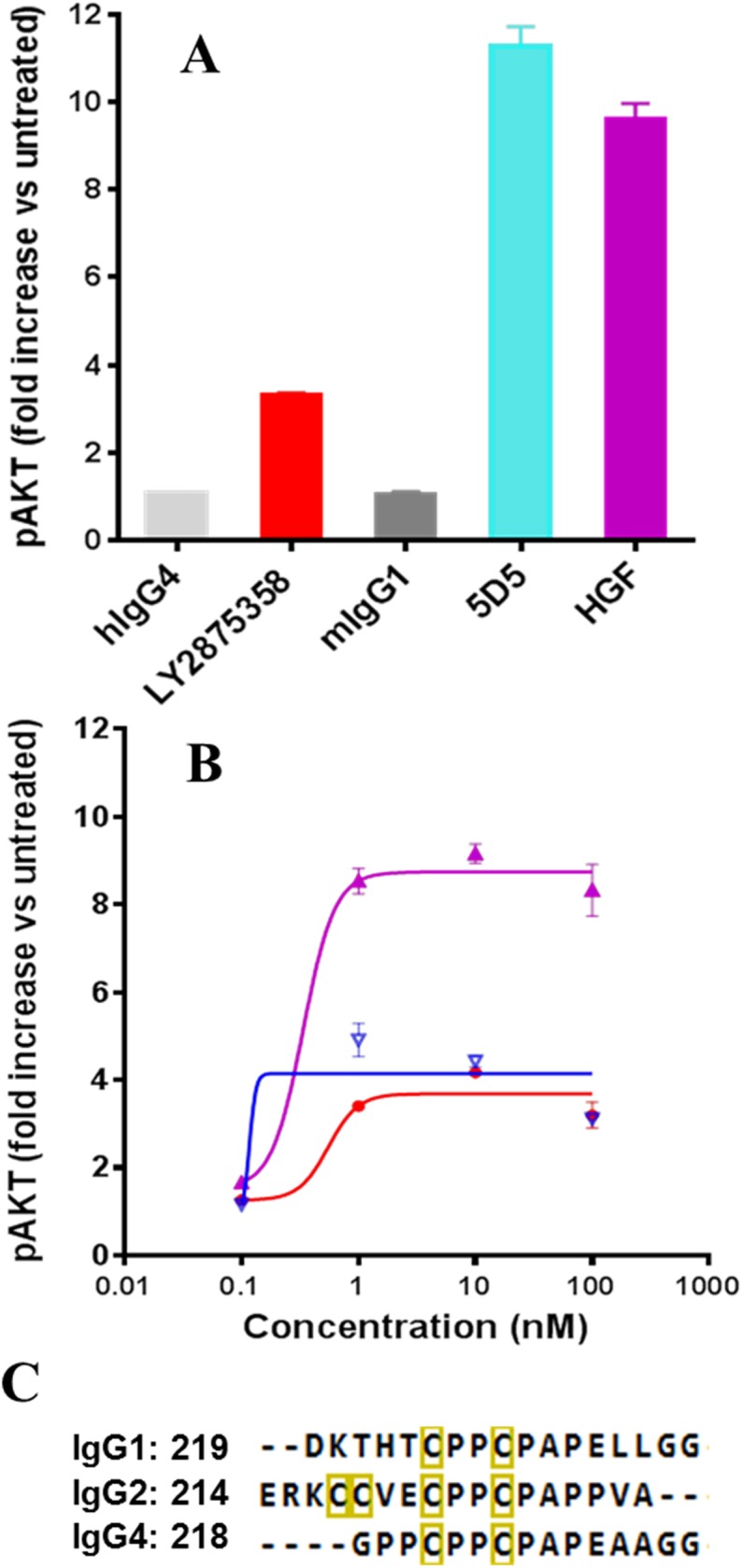
The effect of MET antibody isotypes on pAKT in Caki-1 cells. (A) The humanized IgG4 MET antibody (LY2875358), hIgG4 and mIgG1 induce weak phosphorylation of pan-AKT as compared to the strong phosphorylation of pan-AKT by agonist antibody 5D5 and HGF [12]. (B) Comparison of IgG1 (purple), IgG2 (blue) and IgG4 MET antibodies (red). (C) The sequences in the hinges. The numbering of the first residue is shown in each sequence. The inter-heavy-chain disulfide bonded cysteine residues are indicated in yellow boxes.

We have previously reported [12] multiple *in vitro* bioassays to characterize the agonist properties of LY2875358, using HGF and agonist bivalent MET antibody 5D5 as positive controls with Phospho-AKT as the most sensitive agonist assay. Here we established that LY2875358 (IgG4 isotype) induced only a weak and transient phosphorylation of pan-AKT upon binding to MET (Fig 2A), and this weak phosphorylation of pan-AKT did not stimulate biologic activity in seven functional MET agonist assays [12]. However, the higher levels of AKT phosphorylation induced by MET antibody 5D5 and HGF (Fig 2A) correlated well with cell proliferation, mobility and anti-apoptosis in the same functional assays [12]. We further compared IgG1, IgG2 and IgG4 MET antibodies in the phospho-AKT assay, showing that IgG1 MET antibody significantly increased phospho-AKT activity levels by more than eight-fold, close to the levels from agonist 5D5 and HGF. In the same assay, the IgG2 isotype displayed a slight increase in phosphorylation of AKT as compared to the IgG4 isotype (Fig 2B). Because these antibodies have shown comparable binding affinity to the MET extracellular domain (ECD) by Biacore and have nearly identical sequences except in the hinges (Fig 2C), we hypothesized that the hinge flexibility of antibodies of different isotype might contribute to how antibodies engage MET on the cell surface, hence impacting the agonistic activity.

### Reconstruction of 3D domain conformation models and their relative diversity

**Fig 3:**
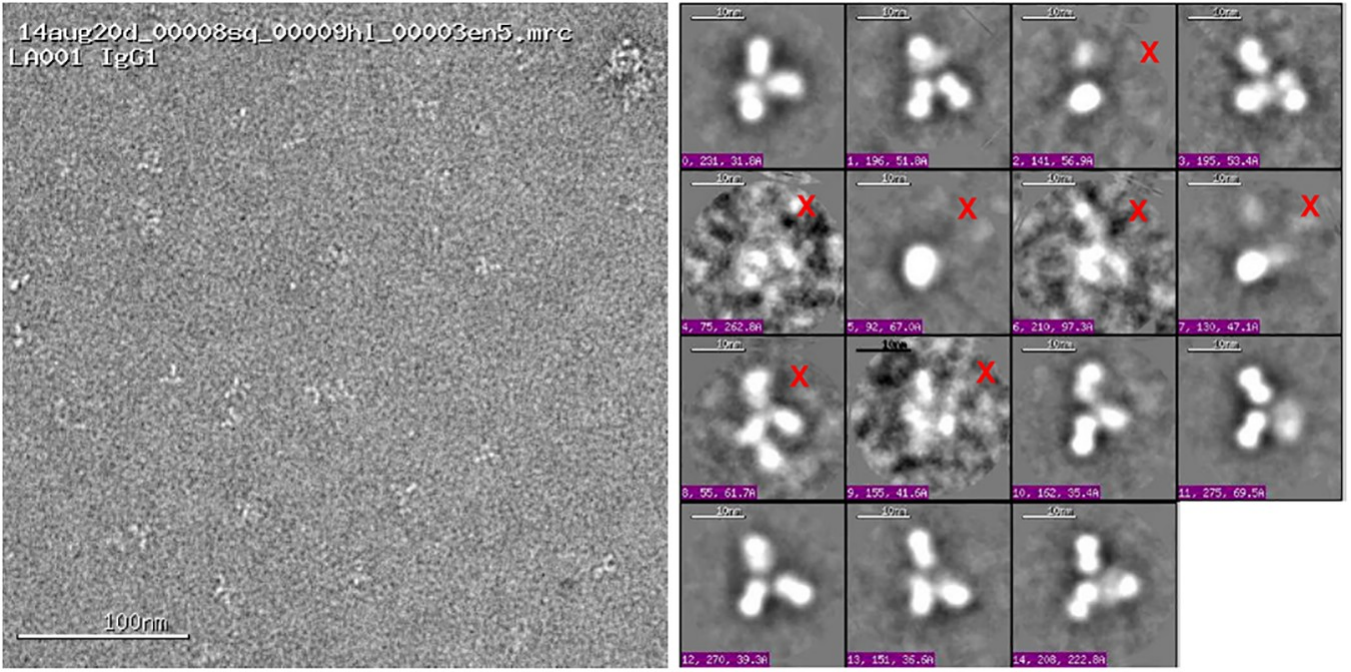
Examples of EM image thumbnails. An EM micrograph with a number of IgG1 particles (left) and EM 2D class average images (right). Not all 2D class averages contained recognizable Y/T-shaped antibody particles; those marked by a red “X” were not used in modeling.

**Fig 4:**
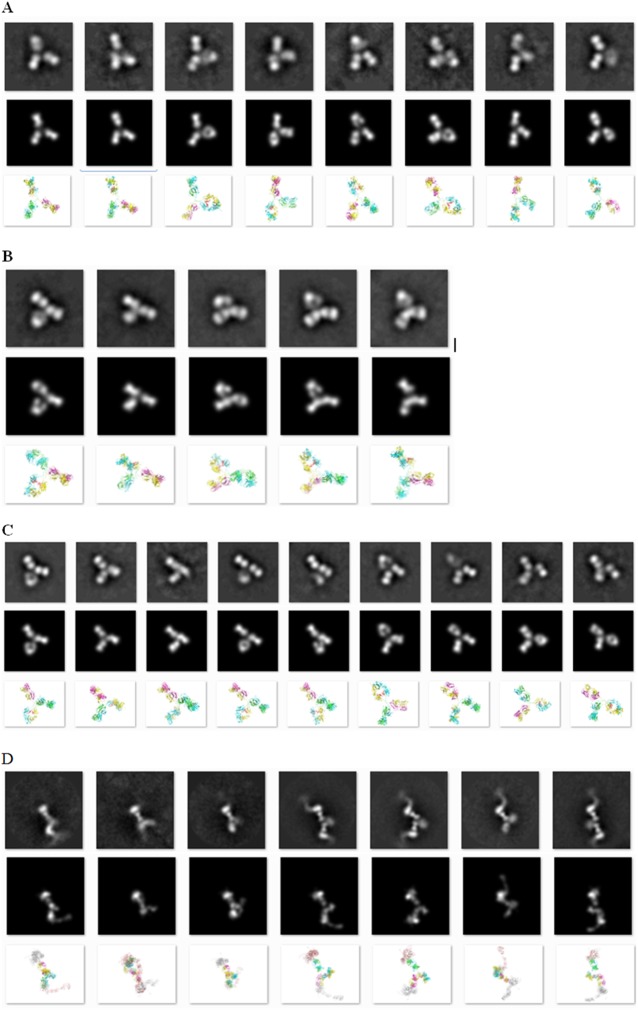
Observed class averages, resulting 3D models, and class averages computed from the models. Images of observed 2D class average (first row), class averages computed from the resulting 3D models (second row) and the ribbon views from the 3D model (light chains in green and magenta, heavy chains in yellow and cyan, glycoside heavy atoms in red), and the models (second row). (A) IgG1: image dimension 160x160 pixels, 2.0 Å/pixel. (B) IgG2: image dimension 160x160 pixels, 2.0 Å/pixel. (C) IgG4: image dimension 106x106 pixels, 3.0 Å/pixel. (D) IgG4-MET antigen complex: image dimension 160x160 pixels, 3.24 Å/pixel.

**Fig 5:**
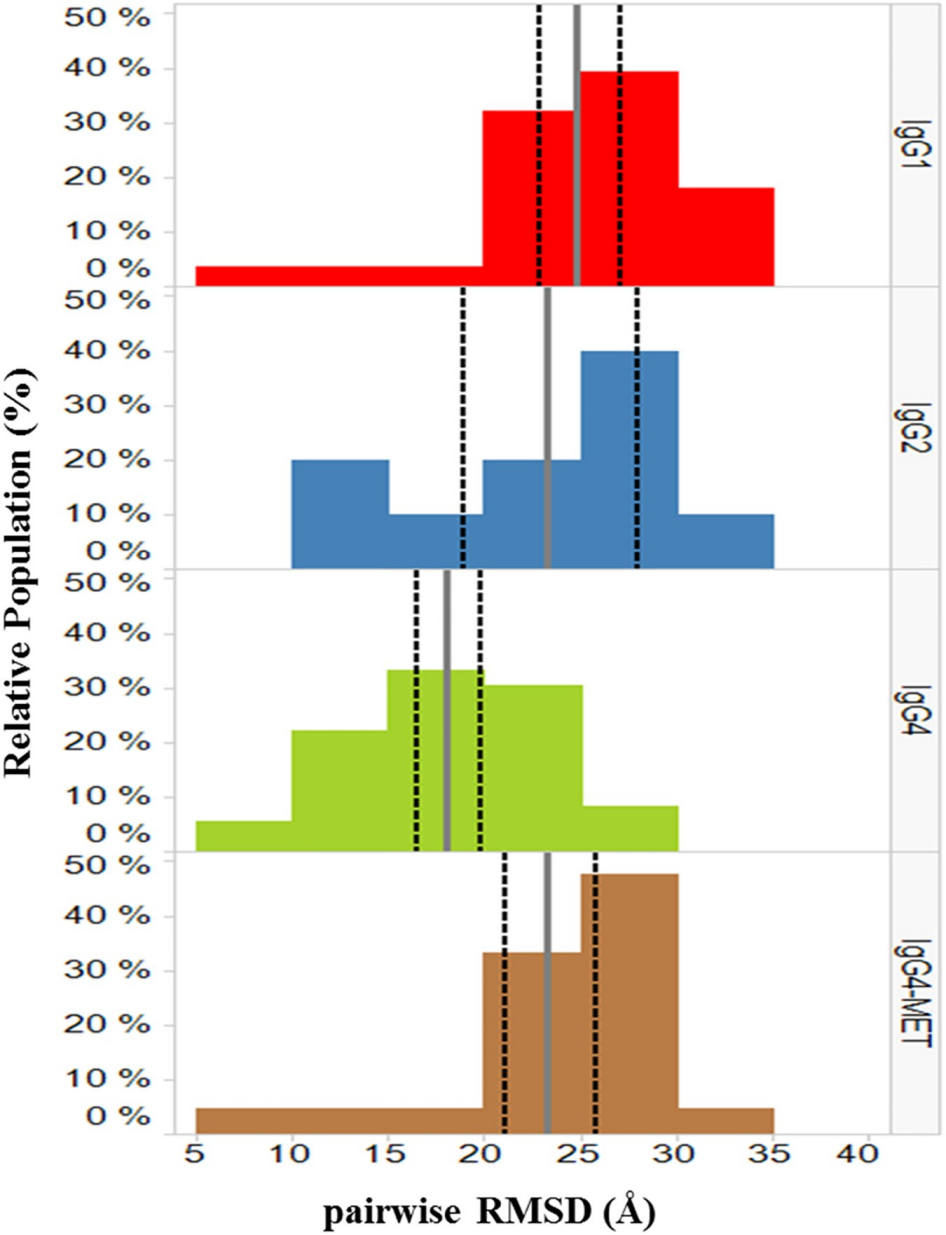
Distributions of pairwise RMSD values for IgG1, IgG2, IgG4 and IgG4- MET complex models. The average values of pairwise RMSD are indicated by the gray vertical solid bars. The 95% confidence intervals of the average are indicated by the black vertical dashed bars. The plots were created using TIBCO Spotfire 6.5.3.[16]

Fig 3 shows an example of EM particle images and their 2D class averages for the IgG1 MET antibody construct. We found that 2D class averages of antibody samples often had the Y/T shape, although in some cases two of the “arms” could be very close to each other. Therefore, we excluded the class averages with images that didn’t display the Y/T shape. Fig 4 shows the observed good quality 2D class average, the corresponding best scoring simulated 2D images, together with the ribbon diagrams of corresponding 3D domain conformation models for all four antibody samples. Eight (IgG1), five (IgG2), nine (IgG4) and seven (IgG4-MET antigen complex) domain conformations were obtained with our protocol. The modeled conformations are quite different within each construct, with pairwise RMSD values ranging from 5.7 to 37.0 Å (Table B in S1 File and Fig 5).

**Fig 6:**
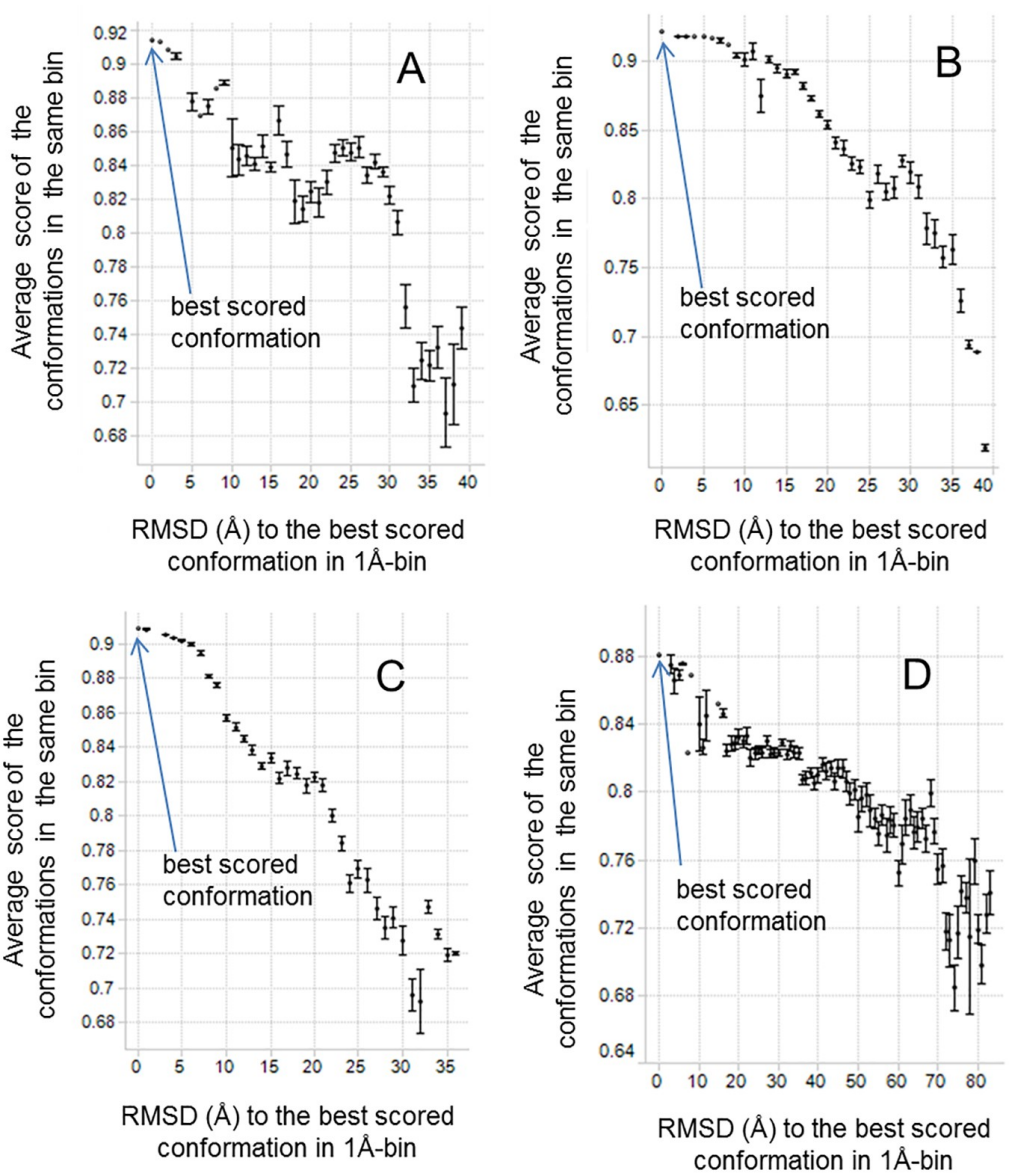
EM2D scores of all conformations and their RMSD values to the highest scoring conformation. The conformations are binned into groups of 1 Å size according to the RMSD values. The standard error is shown as the error bar for each bin. The samples are (A) IgG1, (B) IgG2, (C) IgG4, and (D) IgG4 antigen complex.

EM2D scores (Fig 6) and the visual examination (as described in Materials and Methods) indicated that only the best scoring conformation and its close conformational analogs (within 5 Å RMSD) provided satisfactory representation of the 2D class average. Other domain conformations (more distant than 5 Å RMSD from the best scoring one) had lower EM2D scores and less satisfactory matches to the 2D class average. This observation suggested that the 3D domain conformational models within 5 Å RMSD from the best scoring model were a good explanation of a given 2D class average. It further suggested that the model precision is approximately 5 Å RMSD. This relatively high “model precision” demonstrates that our modeling protocol adds significant structural information to the initial 2D images, which were produced at ~20 Å image resolution.

Approximately 75% of 2D class averages were excluded from our analysis, mostly due to the lack of recognizable Y/T shape (see Fig 3 for the examples of the excluded images) or because we could not find a model matching the class average (Fig 4; Materials and Methods.) These exclusions likely stemmed from the sample preparation and imaging process. Our protocol, while offering model precision of 5 Å RMSD, could only be used to create 3D models for domain conformations that have clearly recognizable antibody shapes in 2D images. As a result of this process limitation, our comparison of domain conformations only focused on the 2D class averages that have recognizable stems and arms of an antibody, potentially resulting in an underestimation of conformational flexibility.

The IgG1 isotype had the highest internal diversity of 3D domain conformations, with average pairwise RMSD of 24.8 Å (Fig 5 and Table 1). The other two MET constructs, IgG2 and IgG4, had lower conformational diversity with average RMSD of 23.3 and 18.0 Å, respectively. The difference of the internal diversity between IgG1 and IgG4 is statistically significant at the 95% confidence level (Fig 5). Both IgG1 and IgG4 have two hinge disulfide bonds, whereas IgG2 has four disulfide bonds. The hinge length is 17 residues for both IgG1 and IgG2, and 15 for IgG4. Both the longer hinge length and lower number of disulfide bonds are expected to lead to higher conformational diversity. The internal conformational diversity observed in our 3D models is more consistent with the increased hinge length than with the number of hinge disulfide bonds.

**Table 1:** Flexibility of domain arrangements. The differences in average values of the domain angles are not statistically significant at 95% confidence level (Figure B in S1 File).

	IgG1	IgG2	IgG4	IgG4-MET
**Hinge**				
number of residues	17	17	15	15
number of disulfide bonds	2	4	2	2
**EM Experiments**				
magnification ratio	110,000	110,000	110,000	67,000
number of recognizable particles	1,688	2,557	4,685	4,000
number of 2D class averages	8	5	9	7
**Domain Angles from 3D Structures (°)**				
average Fab1-Fab2 (population weighted)	114 (114)	139 (139)	138 (140)	139 (144)
average Fab1-Fc (population weighted)	113 (115)	101 (99)	95 (95)	92 (90)
average Fab2-Fc (population weighted)	130 (129)	119 (121)	122 (121)	122 (118)
range of Fab-Fab	82–139	118–164	108–165	108–177
span of Fab-Fab	57	46	57	69
range of Fab1-Fc	71–135	72–142	71–127	63–127
span of Fab1-Fc	64	70	56	64
range of Fab2-Fc	108–163	85–138	104–163	83–154
span of Fab2-Fc	55	53	59	71
**Pairwise RMSD of 3D Structures (Å)**				
Average ±sd	24.8 ±5.7	23.3 ±7.3	18.0 ±5.0	23.3 ±5.6
Median	26.1	24.1	18.1	25.0
Minimum	8.3	11.4	5.7	8.0
Maximum	33.8	34.5	27.2	32.5

In contrast, the number of the modeled conformational states (as opposed to internal conformational diversity) increases as the number of disulfide bonds decreases. A lower number of disulfide bonds resulted in more 2D class averages and a higher number of the corresponding modeled 3D domain conformations. For example, only five unique conformational states were observed for the four-hinge-disulfide-bonded IgG2, while eight and nine conformational states were found for the two-hinge-disulfide-bonded IgG1 and IgG4, respectively.

The angular range of domains relative to one another [2], i.e., the angles between two Fab domains and the angles between the Fab and Fc domains (as defined in Fig 1B), are another commonly used characteristics of antibody flexibility. These parameters, together with other metrics characterizing the modeled constructs, are shown in Table 1 and Figure A in S1 File. Overall, IgG2 and IgG4 constructs had similar average values of all three domain angles (Fab-Fab and two Fab1/2-Fc) while IgG1 and IgG4 had a similar span of the values. The narrower span of the Fab-Fab angles from IgG2 (46 degrees) than that for IgG1 and IgG4 (57 degrees for both) is consistent with the lower expected hinge flexibility of IgG2.

### 3D models for antigen bound IgG4 MET complex

The EM images of the complex between the MET antigen and IgG4 allowed us to compute seven models of domain conformations. While the antigen presence did not change the average values of the domain angles, it led to a larger span of these angles and higher pairwise RMSD values (Table 1 and Fig 5) than found of IgG4 on its own. The domain conformations of the complex were also significantly different from those of the isolated IgG4 with an average pairwise RMSD of 25.7 ±4.2 Å (Table C in S1 File). This observation suggests that angular measurement alone is not sufficient to determine 3D domain conformational differences. The model differences between the antigen-bound and isolated IgG4 indicate that the conformational space of the free form might differ from that for the antigen bound form.

## Discussion and conclusion

It has been shown that antibodies can adopt multiple conformational states. For example, as many as 120 different conformations were reported for the mouse IgG1 from an IPET study [2]. Our results support the existence of multiple conformational states for all our MET antibody constructs. In particular, we found that the IgG1 construct had much wider diversity of domain conformations (Fig 5 and Table 1) and showed the smallest angle between the two Fab domains (Table 1). This behavior is in contrast to the other two isotypes (IgG2 and IgG4) that showed little or low agonist activity. It is tempting to speculate that the agonist activity of MET IgG1 (Fig 2) is related to the extra domain conformational space and/or smaller Fab-Fab angles. Further structural characterization on additional samples with a range of biological agonist profiles will be required to substantiate our hypothesis about the MET antibody agonist activity. Although these results may not be generalizable to all antibodies, our protocol can be readily applied to study dynamics and/or heterogeneity of other antibody constructs.

Our results also indicate that antigen free IgG4 MET conformations are different from those observed in the complex with the antigen. This observation suggests the conformational states required for MET antigen recognition might be different from those accessible to the free antibody. Therefore, the complexes rather than free antibodies are more suited to study structures relevant to the biological activity. Our results demonstrate that the conformational domain variability in our models was dependent primarily on the hinge length, while the number of conformational states revealed by 2D class averages depends on the hinge disulfide patterns.

It is commonly understood that negative staining may introduce artifacts, such as particle flattening [17]. Our analysis is clearly limited by any such artifacts. However, it seems conceivable that staining does not significantly affect conformational variability in the sample, because antibody adherence to the carbon coated grid likely traps the observed conformations prior to application of the negative stain. Also, negative staining with uranyl formate was reported to fix protein samples so rapidly that the overall protein conformations or protein complex formation were not affected [17]. Indeed, the degree of flexibility we observed is consistent with other studies of antibody conformation [2].

The resolution of 2D images and the resulting class averages is lower when compared to the near atomic resolution from the state-of-the art single particle cryo-EM reconstruction [18–24]. But for some applications, in particular to systems containing multiple functional states, the practicality of obtaining single-particle reconstructions at atomic resolution might be time and cost prohibitive. In such cases, our approach may offer a reasonable and economical start before pursuing the more involved experiments. In recent review articles [25, 26], it was estimated that higher resolution data comes at a cost of UK₤1,000/day for cryo-EM-related computing works, in addition to US$5 million instrument cost and half million dollars only for annual operational expenses. A number of studies reported the use of 2D class averages to investigate protein conformation flexibility [27, 28]. As cryo-EM methods become more routinely applicable to particles smaller than 150 kDa, it will be informative to compare cryo-EM class averages of antibody conformations in vitreous ice with those observed by single particle and tomographic analyses of negative-stained samples at similar magnification on the same EM camera.

Our integrative experimental and computational approach was able to provide models of four MET antibody systems with 5 Å RMSD precision. It is expected to be applicable to the determination of low-resolution 3D models of many other antibodies and for testing certain structural hypothesis at a fraction of cost needed for single-particle reconstruction. While 2D images likely reveal only a fraction of all conformational states, they can serve as a good starting point to understanding a relative structural diversity of a particular system. Therefore, lower resolution 2D imaging and resulting class averages could be complementary to 3D Cryo-EM for biological systems that have multiple conformations differing by more than 5 Å RMSD, such as in the case demonstrated here for the MET antibodies.

## Materials and methods

### Antibody samples and preparation

The variable regions from LY2875358 [12] were cloned into human IgG1 and IgG2 constant regions. The antibodies with human IgG1, IgG2 and IgG4 were expressed in Chinese Hamster Ovary cells and purified to greater than 95% homogeneity using Protein A followed by preparative Size Exclusion Chromatography. Human MET ECD containing a FLIS tag was expressed and purified from Chinese Hamster Ovary cells. The IgG4 antibody in complex with human MET ECD antigen was prepared by Size Exclusion Chromatography.

Samples of IgG1, IgG2, IgG4, and IgG4 MET antigen complex were diluted 1:500 with HBS-N pH 7.4 prior to imaging. The samples were then prepared using continuous carbon grid method. Grids were nitrocellulose supported 400-mesh copper. The samples were prepared by applying 3 μL of sample suspension to a cleaned grid, blotting away with filter paper, and immediately staining with Uranyl Formate.

### Phosphorylation of pan-AKT assay

Caki-1 Cells were starved overnight in serum-free medium with 0.5% BSA and then treated with various doses of MET antibodies for 15 minutes. Cell lysates were analyzed for phosphorylation of pan-AKT by MSD ELISA. HGF and agonist bivalent MET antibody 5D5 were used as positive controls.

### EM imaging

EM experiments were performed [29] using an FEI Tecnai T12 electron microscope, operating at 120 keV equipped with an FEI Eagle 4k x 4k CCD camera. Negative stain grids were transferred into the electron microscope using a room temperature stage. Images of each grid were acquired at multiple scales to assess the overall distribution of the specimen. After identifying potentially suitable target areas for imaging at lower magnifications, high magnification images were acquired at nominal magnifications of 110,000X (0.10 nm/pixel) or 67,000X (0.16 nm/pixel). The images were acquired at a nominal underfocus of -2 μm (110,000X) or -3 μm to -2 μm (67,000X), and electron doses of ~25–40 e/Å^2^.

### Integrative modeling

**Fig 7:**
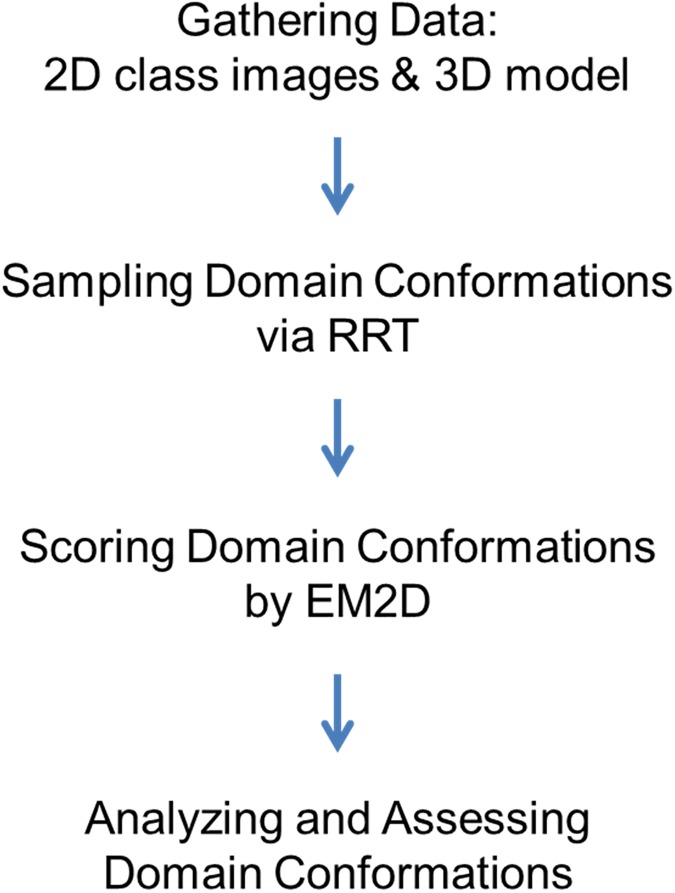
Flowchart of integrative multi-state modeling.

Our integrative structure modeling proceeds through four stages [10, 11]: (1) gathering the data, (2) sampling the domain conformations, (3) scoring the domain conformations, and (4) analyzing and assessing the domain conformations (**Fig 7**):

### Stage 1: Gathering the data for the initial models

#### EM 2D class averages

The individual particles were identified in the high magnification images prior to the alignment and classification. The individual particles were then selected, boxed out, and individual sub-images were combined into a stack to be processed using a reference-free classification method [30]. Individual particles in the 67,000X or 110,000X high magnification images were selected using automated picking protocols [31]. An initial round of alignments was done on each sample, followed by selecting recognizable particles for additional rounds of alignment. Only classes with three recognizable domains were kept for further analysis and 3D structure modeling. Particle alignment and classification were carried out using a reference-free alignment strategy based on the XMIPP [30] processing package. Algorithms in this package aligned the selected particles and sorted them into self-similar groups of classes.

#### Antibody comparative modeling

Initial comparative structure models of the antibody constructs were built by MOE modeling package [6] using an X-ray crystal structure (PDB code 1IGT) [3] as a template. In all cases, disulfide bridges were added (if not created automatically). The structures were then minimized using the Amber10:EHT force field [6] to avoid atomic clashes and resolve any strain created by disulfide addition. The glycosides were also grafted from the 1IGT template structure. The models were then minimized with restraints for non-hydrogen atoms to their original positions, and served as starting points for hinge flexibility exploration. The antibody-antigen complex structure for IgG4 was constructed by grafting the aligned MET-Fab complex (PDB code 4K3J) [32] onto the comparative model of MET IgG4, using the Fab domains’ backbones for superposition. In addition, the complete structure of the MET antigen (PDB code 2UZY) [33] was grafted to the existing model based on the alignment of the overlapping MET region. The final model of the complex was then energy minimized with backbone restraints to avoid the clashes produced by superposition.

### Stage 2: Sampling domain conformation

We used the Rapidly exploring Random Tree (RRT) algorithm [34, 35] to explore the domain conformational space of the full length antibody, using the optimized comparative model as a starting conformation. A modified version of the RRT algorithm implemented in IMP [10, 11] was used, which sampled the dihedral angles of the protein under the closure constraint to keep the disulfide connections among different chains in the hinge region intact. During the search, two Fab domains and the Fc domain were treated as rigid bodies. Up to 100,000 iterations of RRT were performed, and typically about 2,000 diverse domain conformations were generated. The diversity of the domain conformations was characterized by the RMSD values of C_**α**_ atoms between every pair of generated models as well as the domain angles as defined in Fig 1B. In depth discussion of the sampling exhaustiveness is present in S1 File.

### Stage 3: Scoring domain conformation

#### EM2D scoring function

The EM2D module of the IMP program [9–11] was used to compare all antibody domain conformation models to every experimental 2D class average. The 2D class average resolution was estimated to be about 20 Å based on the simulation results. Specifically, the EM2D score of a 3D model reaches a maximum when the model is projected to generate 2D images using the resolution that matches the actual resolution of the 2D class average. We tested a range of resolution values from 2–30 Å and found that the maximal EM2D scores occurred at about 20 Å, thus defining the resolution of the 2D class averages. For every experimental 2D class average, 1,000 different orientations of every domain conformation were projected onto the 2D class image plane to produce the simulated images. The simulated images were then optimized and scored based on the Gaussian-weighted cross-correlation coefficient (i.e., EM2D score) [9] between the observed and the simulated 2D images. For every domain conformational model, the score, simulated 2D image, and parameters of the best-scored orientation were recorded as the final solutions. The PDB file of the conformational model in the final orientation was generated along with the protein ribbon-view image created using a PyMOL script [36].

A scoring function that can rank alternative models by their accuracy is an essential part of any structure modeling. Good scoring functions can correctly rank models over a broad range of RMSD values from the native state. We examined whether or not such “scoring funnels” existed for EM2D scoring [37] and determined their shape for the specific antibody constructs. The EM2D scores of all RRT generated candidate domain conformations are plotted against the RMSD values from the highest scoring model in Fig 6. When fitting to the same experimental image, differences were often observed from two simulated images even when the EM2D scores differed by only 0.01. We thus expanded the score threshold window to 0.03 (from the best scoring one) to broaden the pool of candidates for the visual inspection and final model refinement.

### Stage 4: Analysis and assessment of the ensemble

For every experimental 2D class average, all domain conformational models were sorted in descending order by their EM2D scores (see Stage 3) and clustered by a “leader” algorithm [38, 39] to facilitate visual examination. In depth discussion on the EM2D scoring as it relates to image selection is present in S1 File. The highest scoring domain conformation was selected as the first leader object and removed from the list. The all-residue Cα RMSD values of every subsequent domain conformation in the sorted list to the already-found leader conformation determined whether the conformation was designated as a new leader or a member of an existing cluster represented by a previously-selected leader. A new leader conformation was selected with the highest EM2D score and the RMSD to existing leaders exceeding the threshold value. Four thresholds of the RMSD values were used for the leader clustering, 5, 10, 15, and 20 Å. For each threshold, up to 20 top scoring leaders and several other top scoring cluster members were visually examined for their fit to the experimental 2D class average.

The visual inspection, aided by PyMOL [36] protein ribbon representation, was used only to select the cutoff for the EM2D score that ensures consistency between the 2D class average, the simulated image, and the 3D model ribbon view, including the assignment of the Fc domain. Specifically, visual inspection of matches and mismatches confirmed that the EM2D score can indeed be used for ranking models based on class averages. However, this visual examination also suggested that some 3D models with slightly lower EM2D scores also match the 2D class averages well, if these models were within 5 Å RMSD to the best scoring model, resulting in the estimate of model precision of 5 Å RMSD. Visualization further revealed that the EM2D score of at least 0.83 was needed to ensure such consistency; otherwise, mismatches between the models and the class averages could occur due to similar shapes of the three domains. Therefore, we elected to discard the class averages without models with the EM2D score of at least 0.83. In summary, a good fit between an experimental 2D class average and a 3D antibody domain conformation has two attributes: (1) EM2D score of at least 0.83; and (2) the observed 2D image, the simulated 2D image, and the ribbon view image are consistent with each other in the overall shape and Fc domain assignment.

The final 3D structures representing the best matches to the 2D class averages were subsequently transferred to the MOE modelling package [6], and minimized with constraints for Fab and Fc domain backbone heavy atoms while allowing the hinge atoms to be flexible. The glycosides were attached to the glycosylation sites on the Fc domain heavy chains. The resulting minimized structures were transferred into Maestro15.3 [40], optimized with the protein preparation wizard and solvated in orthorhombic TIP3P water box for the NPT Molecular Dynamics (MD) local refinement with Langevin thermostat and PME using Desmond package [40]. The refinement protocol consisted of three steps of constrained minimization: 5,000 steps with heavy atom restraints, followed by unrestrained 50,000 steps, restrained heating at 30 ps at temperature 100 K, 200 K and 300 K equilibration. The 5 ns MD production run for each 3D structure with backbone heavy atom constraints for Fab and Fc domains allowed for equilibration of side chains, glycosides and hinge residues. The resulting structures were then minimized with 1,000 steps of steepest descent minimization to produce the final 3D models.

## Supporting information

S1 File Diversity of candidate conformation states for quality reconstructions. The file also contains two figures and three tables.
(DOCX)

S2 File The atomic coordinates of all 3D structure models in PDB format. (ZIP)
