# Supplementary material for: Reconstruction of 3D structures of MET antibodies from electron microscopy 2D class averages
Source: PLoS One. 2017 Apr 13;12(4):e0175758. doi: 10.1371/journal.pone.0175758 (PMC5391116; doi:10.1371/journal.pone.0175758)
Supplement: S1 File — The file also contains two figures and three tables. (DOCX) [file pone.0175758.s001.docx]

**Supporting Information**

**Reconstruction of 3D Structures of MET Antibodies from EM 2D Class Averages**

Qi Chen^1*^, Michal Vieth^1*^, David E. Timm^1^, Christine Humblet^1^, Dina Schneidman-Duhovny^2^, Ilan E. Chemmama^2^, Andrej Sali^2^, Wei Zeng^1^, Jirong Lu^1^, Ling Liu^1^

Lilly Research Laboratories, Eli Lilly and Company, Indianapolis, Indiana, 46285, United States of America

^2^ Department of Bioengineering and Therapeutic Sciences, Department of Pharmaceutical Chemistry, and California Institute of Quantitative Biosciences, University of California, San Francisco, California, 94158, United States of America

* Corresponding Authors: chen_qi_qc@lilly.com (QC), vieth_michal@lilly.com (MV)

## Diversity of candidate conformations for quality reconstructions

We found, based on both EM2D score and visual inspection, that a single structural model such as an X-ray crystal structure (e.g., PDB code 1IGT [1]) leads to significantly lower scores than those obtained from RRT generated diverse domain conformational models (Table A). This observation is consistent with the broad conformational space observed in X-ray crystal structures of different IgG isotypes (Fig. 1) and diverse conformational space reported from IPET experiments for IgG1 [2]. On average, the EM2D scores for any of the X-ray structures were at least 0.05 lower than those for the best scoring conformations from our multi-conformation RRT protocol.

Fig A further highlights the importance of using a large, diverse conformational ensemble to identify the models producing highest scoring simulated images. The highest score could be found only after a certain number of conformations were explored with RRT sampling. For three full length antibody samples, the highest scoring conformations were found after exploring 400-1000 diverse conformations. For the IgG4 antigen complex, 600-2,000 conformations were needed. To ensure appropriate coverage, in all our reconstructions we generated at least 1,300 diverse conformations for antibody alone, and >2,300 for the antibody-antigen complex.


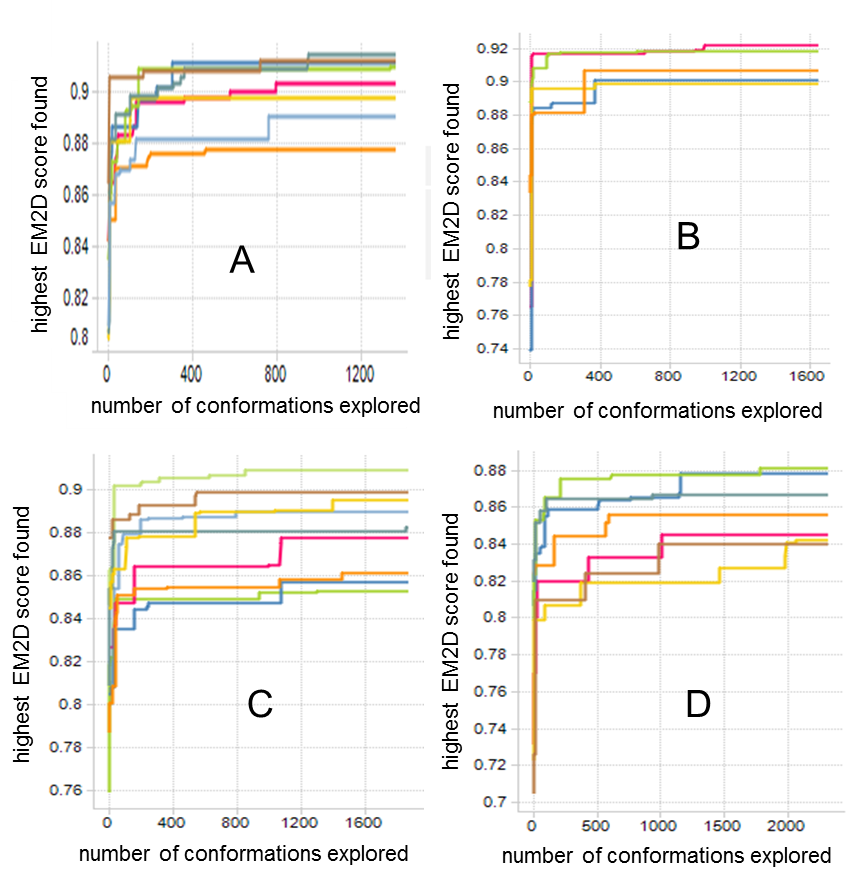


**Fig A**. **The highest scores found vs. number of conformations explored and scored by EM2D**. Each line represents one 2D class average, lines are colored by the 2D class averages. The conformations are sorted in the order of being generated by RRT conformational search. (A) IgG1, (B) IgG2, (C) IgG4, and (D) IgG4 antigen complex.

**
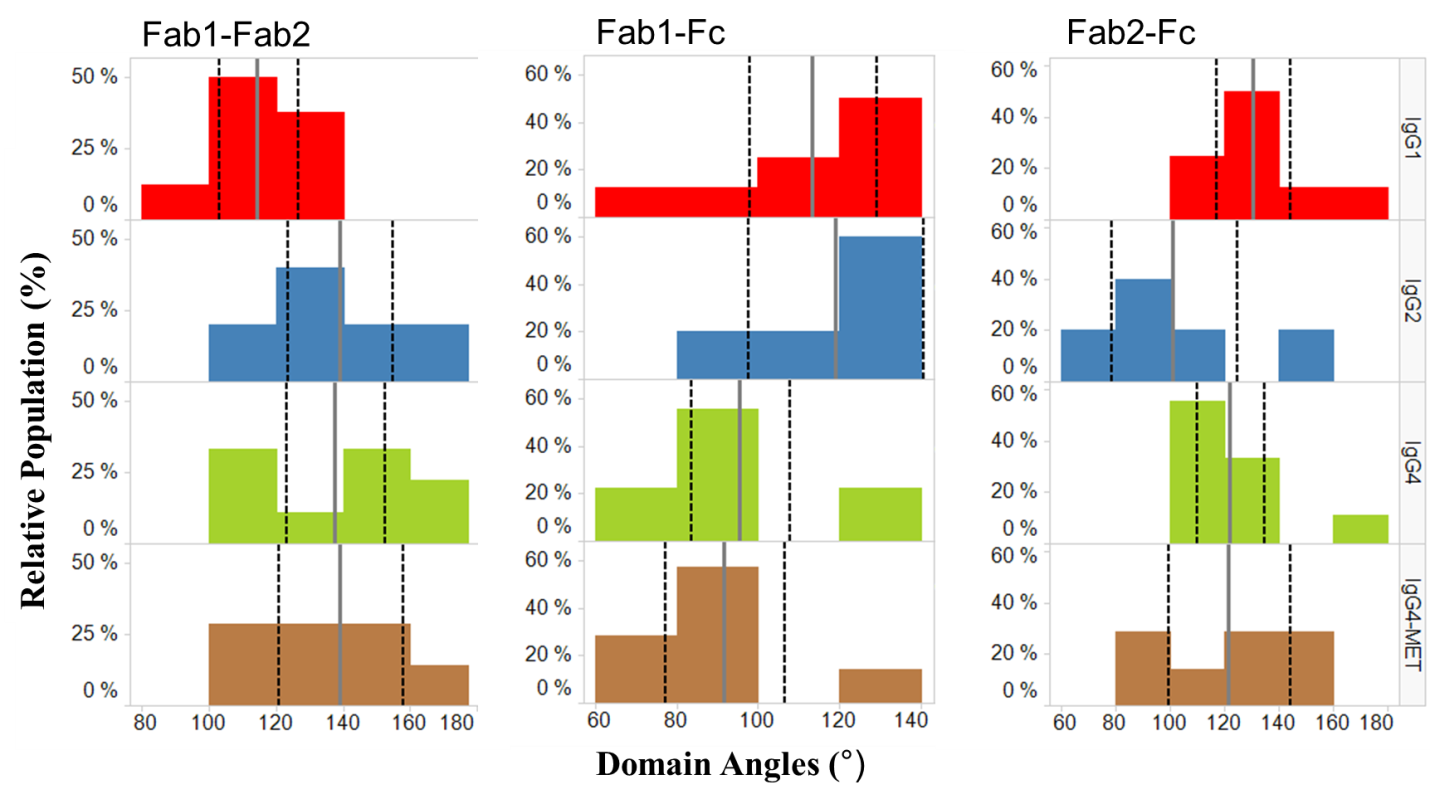
**

**Fig B. Distributions of the domain angles measured from 3D models**. The average value of pairwise RMSD is indicated by the gray vertical solid bars. The 95% confidence intervals of the average are indicated by the black vertical dashed bars. The plot was created using TIBCO Spotfire software 6.5.3. [3]

**Table A. Number of particles of 2D class averages and the best EM2D scores from the best models and the single model of an X-ray crystal structure**

| **sample** | **2D class average** | | **EM2D score from** | |
| --- | --- | --- | --- | --- |
|  | **ID** | **number of particles** | **best conformation** | **1igt** |
| IgG1 | 1 | 270 | 0.914 | 0.8477 |
|  | 2 | 151 | 0.9119 | 0.8269 |
|  | 3 | 208 | 0.9109 | 0.8235 |
|  | 4 | 231 | 0.9089 | 0.8845 |
|  | 5 | 196 | 0.9027 | 0.8258 |
|  | 6 | 195 | 0.8976 | 0.8855 |
|  | 7 | 162 | 0.8899 | 0.8597 |
|  | 8 | 275 | 0.8772 | 0.8014 |
| IgG2 | 1 | 483 | 0.9219 | 0.8906 |
|  | 2 | 440 | 0.9186 | 0.8849 |
|  | 3 | 539 | 0.907 | 0.8416 |
|  | 4 | 624 | 0.9009 | 0.8448 |
|  | 5 | 471 | 0.8988 | 0.8302 |
| IgG4 | 1 | 790 | 0.9093 | 0.8224 |
|  | 2 | 429 | 0.8992 | 0.8569 |
|  | 3 | 348 | 0.8952 | 0.8804 |
|  | 4 | 716 | 0.8897 | 0.8437 |
|  | 5 | 491 | 0.8825 | 0.8146 |
|  | 6 | 551 | 0.878 | 0.803 |
|  | 7 | 540 | 0.8611 | 0.8106 |
|  | 8 | 378 | 0.8573 | 0.7571 |
|  | 9 | 442 | 0.8526 | 0.836 |
| IgG4-cMet | 1 | 371 | 0.8811 |  |
|  | 2 | 296 | 0.8785 |  |
|  | 3 | 408 | 0.8664 |  |
|  | 4 | 877 | 0.8555 |  |
|  | 5 | 939 | 0.8452 |  |
|  | 6 | 440 | 0.8422 |  |
|  | 7 | 669 | 0.8403 |  |

**Table B. Pairwise RMSD comparison of models from different antibodies**

|  |  | IgG1 | | | | | | | | IgG2 | | | | |
| --- | --- | --- | --- | --- | --- | --- | --- | --- | --- | --- | --- | --- | --- | --- |
|  | model | 1 | 2 | 3 | 4 | 5 | 6 | 7 | 8 | 1 | 2 | 3 | 4 | 5 |
| IgG2 | 1 | 28.2 | 25.8 | 23.1 | 32.9 | 35.7 | 30.5 | 24.7 | 25.8 |  |  |  |  |  |
|  | 2 | 27.9 | 29.9 | 22.5 | 29.5 | 29.2 | 28.9 | 33.4 | 29.8 |  |  |  |  |  |
|  | 3 | 33.5 | 34.6 | 27.5 | 26 | 33.6 | 26.1 | 20.9 | 18.5 |  |  |  |  |  |
|  | 4 | 32.4 | 32.6 | 25 | 23.2 | 30.5 | 21.4 | 23.9 | 15.7 |  |  |  |  |  |
|  | 5 | 31.7 | 32.1 | 24.7 | 25.7 | 26.5 | 23.8 | 26.7 | 13.6 |  |  |  |  |  |
| IgG4 | 1 | 24 | 26.8 | 9.49 | 28.8 | 28.3 | 26.9 | 24 | 22.9 | 22.6 | 21.3 | 30.4 | 26.3 | 24.4 |
|  | 2 | 30.1 | 29.8 | 24.5 | 27 | 25.5 | 23.5 | 30.7 | 23 | 26.1 | 24.2 | 30.9 | 26.2 | 23.6 |
|  | 3 | 27.6 | 26.2 | 17.8 | 29.6 | 32 | 26.8 | 20.9 | 21.9 | 16.6 | 25.8 | 25.8 | 23.2 | 24.6 |
|  | 4 | 28.2 | 31.1 | 10.5 | 30.4 | 31.9 | 28.4 | 25.4 | 25.6 | 22 | 21.8 | 30.3 | 26.7 | 26.9 |
|  | 5 | 27.6 | 29.3 | 17 | 33.2 | 29.8 | 30.6 | 32.5 | 29.5 | 25.3 | 22.3 | 36.9 | 31.8 | 29.1 |
|  | 6 | 20.8 | 25.1 | 14.7 | 26.9 | 23.2 | 26.2 | 23.1 | 20.4 | 27.6 | 25.9 | 29.7 | 26.6 | 22.8 |
|  | 7 | 28.6 | 29.6 | 16.2 | 31.9 | 33.7 | 30.4 | 28.1 | 26.3 | 20.3 | 16.6 | 30.8 | 26.8 | 26.6 |
|  | 8 | 23.1 | 26.5 | 13.9 | 26.2 | 24.9 | 25.2 | 21 | 16.9 | 26.2 | 24.6 | 26.7 | 23.5 | 19.9 |
|  | 9 | 28.4 | 29.1 | 19.1 | 34.6 | 28.9 | 32.1 | 33.1 | 27.3 | 28.4 | 20.5 | 37 | 31.4 | 25.8 |

**Table C. Pairwise RMSD Comparison between IgG4 and its antigen complex.**

| IgG4 models | models of IgG4 antigen complex | | | | | | |
| --- | --- | --- | --- | --- | --- | --- | --- |
|  | 1 | 2 | 3 | 4 | 5 | 6 | 7 |
| 1 | 27.9 | 18.1 | 27 | 23.8 | 24.3 | 23.1 | 25.4 |
| 2 | 27.7 | 23.1 | 28.5 | 20.7 | 30.5 | 28.2 | 24.1 |
| 3 | 26.4 | 21 | 23.6 | 22.4 | 21.8 | 23.9 | 25.6 |
| 4 | 28.9 | 22 | 27.1 | 22.6 | 24.8 | 26.5 | 22.8 |
| 5 | 32.8 | 25.6 | 34.3 | 25.3 | 30.7 | 28.7 | 25.9 |
| 6 | 28.2 | 15.6 | 26.2 | 28.5 | 27.1 | 21.1 | 31.9 |
| 7 | 27.1 | 23.2 | 27.8 | 21.1 | 26.1 | 29 | 20.9 |
| 8 | 25.4 | 13.6 | 22.7 | 26.1 | 24.8 | 20.8 | 29.9 |
| 9 | 32.8 | 22.8 | 33.8 | 27.1 | 32.9 | 29.7 | 29.9 |

1. Harris LJ, Larson SB, Hasel KW, McPherson A. Refined structure of an intact IgG2a monoclonal antibody. Biochemistry. 1997;36(7):1581-97.

2. Zhang X, Zhang L, Tong H, Peng B, Rames MJ, Zhang S, et al. 3D Structural Fluctuation of IgG1 Antibody Revealed by Individual Particle Electron Tomography. Sci Rep. 2015;5:9803.

3. TIBCO Software Inc. Available from: http://www.tibco.com.
